# Genotyping *Candida albicans* from *Candida* Leukoplakia and Non-*Candida* Leukoplakia Shows No Enrichment of Multilocus Sequence Typing Clades but Enrichment of ABC Genotype C in *Candida* Leukoplakia

**DOI:** 10.1371/journal.pone.0073738

**Published:** 2013-09-18

**Authors:** Mohammed H. Abdulrahim, Brenda A. McManus, Stephen R. Flint, David C. Coleman

**Affiliations:** 1 Division of Maxillofacial Surgery, Oral Medicine and Oral Pathology, Dublin Dental University Hospital, University of Dublin, Dublin, Republic of Ireland; 2 Microbiology Research Unit, Division of Oral Biosciences, Dublin Dental University Hospital, Trinity College Dublin, University of Dublin, Dublin, Republic of Ireland; Louisiana State University, United States of America

## Abstract

Oral leukoplakias are histopathologically-diagnosed as *Candida* leukoplakia or non-*Candida* leukoplakia by the presence or absence of hyphae in the superficial epithelium. *Candida* leukoplakia lesions have significantly increased malignant potential. *Candida albicans* is the most prevalent fungal species associated with oral leukoplakia and may contribute to malignant transformation of *Candida* leukoplakia. To date, no detailed population analysis of *C. albicans* isolates from oral leukoplakia patients has been undertaken. This study investigated whether specific *C. albicans* genotypes were associated with *Candida* leukoplakia and non-*Candida* leukoplakia in a cohort of Irish patients. Patients with histopathologically-defined *Candida* leukoplakia (n = 31) or non-*Candida* leukoplakia (n = 47) were screened for *Candida* species by culture of oral rinse and lesional swab samples. Selected *C. albicans* isolates from *Candida* leukoplakia patients (n = 25), non-*Candida* leukoplakia patients (n = 19) and oral carriage isolates from age and sex matched healthy subjects without leukoplakia (n = 34) were subjected to multilocus sequence typing (MLST) and ABC genotyping. MLST revealed that the clade distribution of *C. albicans* from both *Candida* leukoplakia and non-*Candida* leukoplakia lesions overlapped with the corresponding clade distributions of oral carriage isolates and global reference isolates from the MLST database indicating no enrichment of leukoplakia-associated clones. Oral leukoplakia isolates were significantly enriched with ABC genotype C (12/44, 27.3%), particularly *Candida* leukoplakia isolates (9/25, 36%), relative to oral carriage isolates (3/34, 8.8%). Genotype C oral leukoplakia isolates were distributed in MLST clades 1,3,4,5,8,9 and 15, whereas genotype C oral carriage isolates were distributed in MLST clades 4 and 11.

## Introduction

Several *Candida* species cause opportunistic infections in humans, the most prevalent and pathogenic of which is *Candida albicans*. *Candida albicans* is also the most common *Candida* species isolated from the oral cavity as both a commensal and pathogenic organism in healthy individuals and those with underlying disease [1].

Oral leukoplakia is used as a general, clinical descriptive term to describe oral white patches or lesions of questionable risk, having excluded other known diseases that carry no increased risk for cancer [2]. These lesions can be further defined based on histopathological diagnosis or clinical history. Lehner [3] introduced the term “*Candida* leukoplakia” to describe chronic oral *Candida* infection presenting in the form of leukoplakia, for which increased malignant potential has been reported [4] compared to simple leukoplakia. *Candida* leukoplakia usually presents clinically as a well demarcated, rough, raised, white plaque-like lesion that cannot be rubbed off. The lesion may have an homogenous white surface, (homogenous leukoplakia), or in contrast a non-homogenous, nodular or speckled apperance, with an erythematous base. The commissure is the most common site affected, but it may also affect the palate and tongue. These lesions may be associated with angular cheilitis. *Candida albicans* is by far the most prevalent species associated with oral leukoplakia lesions [5].

*Candida* leukoplakia (CL) lesions are difficult to distinguish from non-*Candida* leukoplakias (NCLs) clinically, but the presence of invading *Candida* hyphae in the superficial layer of epithelium accompanied by infiltratration of polymorphic neutrophils histologically distinguish CL lesions [4], [6]. The role of *Candida* in inducing keratotic changes and cellular atypia is uncertain, and there is some controversy regarding the initiation of epithelial hyperplasia by *Candida*. Jepsen and Winther [7] suggested that *Candida* invades a pre-existing hyperplastic lesion, whereas in contrast Cawson and Lehner [4] proposed that *Candida* infection is the primary cause of CL. Holmstrup and Besserman [8] demonstrated reversion of non-homogenous CL to the homogenous type after the use of topical antifungal agents. McCullough et al. [9] reported a strong statistical association between increased oral yeast density, oral epithelial dysplasia and oral squamous cell carcinoma, with the degree of epithelial dysplasia correlating with increased oral yeast density. Based on these findings, the authors hypothesised that the progression of *Candida* leukoplakia to dysplasia is promoted by *C. albicans*. With NCL lesions, the inference is that *Candida* species isolated from these lesions are non-pathogenic, commensal yeast blastospore contaminants.

Detailed molecular epidemiological and population studies based on *C. albicans* isolates recovered from CL lesions are, to date, mostly lacking. This is most likely due to practical difficulties in obtaining sufficient isolates from such lesions for detailed studies due to the relative rarity of the condition and the requirement for biopsy and histopathological investigation. Furthermore, histopathological diagnosis of such lesions has previously been quite vague. A previous report based on biotyping experiments suggested that *C. albicans* isolates recovered from oral leukoplakia lesions in 17 separate patients may be genetically different to those which typically colonise the normal oral mucosa [5]. In contrast, another study that used PCR-based fingerprinting methods using two interrepeat primer combinations and a minisatellite-specific primer failed to find clonal restrictions among 38 *C. albicans* strains recovered from 17 patients with CL [10]. However, in this study not all lesions were histopathologically confirmed as CL.

Multilocus sequence typing (MLST) of *C. albicans* has been used widely to assess the genetic relatedness of isolates recovered from disparate geographic locations and for *C. albicans* population analysis [11], [12], [13]. MLST is a DNA-based method that examines nucleotide sequence variation in seven housekeeping genes and is highly reproducible between different laboratories. A curated, internet-based *C. albicans* MLST database (http://calbicans.mlst.net) has facilitated the comparison of isolates from a wide variety of anatomical sites and geographic locations. *Candida albicans* isolates can also be ABC genotyped based on the presence or absence of an intron in 25S rDNA [14]. Although this method has previously been described as a helpful confirmatory test in instances where isolates differ by one or more single nucleotide polymorphisms (SNPs) in MLST [15], and has been used to demonstrate geographical and temporal differences amongst *C. albicans* isolates [16], the method examines only one genetic marker, has a low discriminatory power (i.e. discriminates isolates into one of three genotypes) and therefore should not be used as a definitive test for isolate relatedness [15]. Genotypes A and B predominate amongst *C. albicans*, whereas genotype C is quite rare [17].

The purpose of the present study was to characterise the population of *C. albicans* isolates recovered from histopathogically-defined CL and NCL lesions from a cohort of Irish oral leukoplakia patients using MLST and ABC genotyping in order to determine if these lesions could be associated with specific *C. albicans* lineages.

## Materials and Methods

### Ethics statement

Ethical approval for this study was obtained from the St. James's Hospital (SJH) and Adelaide and Meath Hospitals including the National Children's Hospital (AMNCH) Research Ethics Committee, Dublin, Ireland. Prior to enrollment in the study, all participants were provided with comprehensive patient information documentation and all participants included in the study provided written consent. All documentation provided to patients, including consent forms were pre-approved by the Research Ethics Committee. This paper does not include any identifying, or potentially identifying, patient information.

### Study group

Oral rinse and swab samples were collected from 78 patients with oral leukoplakia attending the Oral Medicine and Dysplasia clinics at the Dublin Dental University Hospital (DDUH) between December 2006 and March 2009 (Table 1). Patients were examined and followed-up by two oral medicine consultants and an oral maxillofacial consultant. A full medical and dental history was recorded and a comprehensive oral examination undertaken. A clinical diagnosis of oral leukoplakia was made as described previously [2]. A histopathological diagnosis of CL was made based on the detection of hyphae and hyphal-associated polymorphonuclear leukocytes in CL biopsies. Similarly, the absence of hyphae in leukoplakia biopsies was used to define NCL lesions. Squamous cell carcinoma developed in two CL patients at the lesional site within the follow up two-year period. None of the NCL group developed malignant change to date. The mean age of the patient group was 57.8 years (range 29–87 years) and the sex distribution was 40 (51%) male and 38 (49%) female. Forty-nine patients were smokers (62.8%), 20 of whom were diagnosed with CL. Fifteen (48.3%) of the CL patients and 11 (23.4%) NCL patients wore upper dentures.

**Table 1:** Oral leukoplakia patient details and *Candida* species recovered.

Patient	Sex	Age	Lesionsite	Denture wearer	Smoker	Degree of dysplasia	*Candida* cell density		*Candida* species isolated
**CL**							**Rinse cfu/ml**	**Swab** **cfu**	
**01**	M	58	PAL	Yes	Yes	Moderate	40	420	*C. albicans*
**02**	F	52	BM	Yes	Yes	No	70	35	*C. albicans*
**03**	M	74	TON	Yes	No	Severe	0	415	*C. albicans*
**04**	M	38	BM	No	Yes	Moderate	157	42	*C. albicans*
**05**	M	60	BM	Yes	Yes	Moderate	C	380	*C. albicans*
**06**	F	77	TON	No	No	Mild	1450	120	*C. albicans*
**07**	M	73	AR	Yes	Yes	No	0	72	*C. albicans* *C. glabrata*
**08** a	F	58	GIN	No	No	Severe (SCC)	0	480	*C. albicans*
**09**	F	47	BM	No	No	Severe	0	1525	*C. albicans*
**10**	M	55	BM	Yes	Yes	Mild	2060	130	*C. albicans* *C. dubliniensis*
**11**	F	73	BM	Yes	Yes	Moderate	1201025	8447	*C. albicans* *C. parapsilosis* *C. guilliermondii*
**12**	F	44	BM	No	Yes	Moderate	2	15	*C. albicans*
**13**	M	29	BM	Yes	No	Severe	2256035	44191	*C. albicans* *C. tropicalis* *S. cerevisiae*
**14** a	M	63	BM	No	Yes	Severe (SCC)	SC	166	*C. albicans*
**15**	F	64	BM	No	No	Severe	C	SC	*C. albicans*
**16**	F	57	BM	No	Yes	Severe	450	400	*C. albicans*
**17**	M	41	BM	No	Yes	Moderate	160	10	*C. albicans*
**18**	M	69	BM	No	Yes	Moderate	27	0	*C. albicans*
**19**	F	76	PAL	Yes	No	Severe	SC	200	*C. albicans*
**20**	M	72	PAL	No	No	Moderate	290	0	*C. albicans*
**21**	F	63	TON	No	No	Severe	C	SC	*C. albicans*
**22**	M	77	BM	Yes	Ex	Moderate	0	0	None
**23**	M	45	BM	Yes	Yes	Moderate	10	2	*C. albicans*
**24**	F	60	BM	No	No	Moderate	240	125	*C. albicans*
**25**	M	47	BM	No	Yes	Severe	C130	4406	*C. albicans* *C. glabrata*
**26**	M	63	BM	No	Yes	Moderate	SC	SC	*C. albicans*
**27**	F	60	BM	Yes	Yes	Moderate	280	92	*C. albicans*
**28**	F	46	BM	Yes	Yes	Mild	0	250	*C. albicans*
**29**	M	46	TON	No	Yes	No	293	220	*C. albicans*
**30**	F	40	BM	No	Yes	Moderate	215	185	*C. albicans*
**31**	M	56	BM	Yes	Yes	Mild	0	0	None
**NCL**									
**32**	F	79	BM	Yes	Ex	Severe	20	C	*C. albicans*
**33**	M	55	BM	Yes	Ex	Severe	50	0	*C. albicans*
**34**	M	62	BM	No	Yes	Moderate	0	2	*C. albicans*
**35**	M	46	TON	No	Ex	Moderate	150	0	*C. albicans*
**36**	F	82	AR	Yes	Yes	Moderate	3010	07	*C. albicans* *C. glabrata*
**37**	F	41	BM	No	Yes	Severe	48	18	*C. albicans*
**38**	M	83	TON	Yes	Yes	Moderate	172	150	*C. albicans*
**39**	M	68	PAL	No	Yes	No	4	0	*C. albicans*
**40**	F	39	BM	No	No	Moderate	10	0	*C. albicans*
**41**	M	59	TON	No	Ex	Severe	16	9	*C. albicans*
**42**	F	49	TON	No	No	Severe	SC	0	*C. albicans*
**43**	F	44	BM	No	No	Severe	83	32	*C. albicans*
**44**	M	51	PAL	No	Yes	Moderate	79	8	*C. albicans*
**45**	F	64	BM	No	Yes	Severe	39	10	*C. albicans*
**46**	M	76	BM	No	Yes	Mild	10	9	*C. albicans*
**47**	F	55	AR	No	Yes	Mild	50	11	*C. albicans*
**48**	M	40	AR	No	Yes	Mild	0	3	*C. albicans*
**49**	F	35	FM	Yes	Yes	Moderate	485113	01	*C. albicans* *C. krusei*
**50**	F	65	BM	No	Yes	Moderate	20	0	*C. albicans*
**51**	M	67	BM	Yes	Yes	Moderate	0	0	None
**52**	F	62	FM	No	Yes	Moderate	100	0	*C. albicans*
**53**	M	73	GIN	No	Yes	Moderate	0	0	None
**54**	M	58	FM	No	Ex	Moderate	110	13	*C. albicans*
**55**	M	78	BM	No	Yes	Moderate	40	12	*C. albicans*
**56**	F	51	BM	No	Ex	Moderate	0	203	*C. albicans*
**57**	F	55	TON	Yes	No	Severe	260	107	*C. albicans*
**58**	F	87	AR	Yes	No	Mild	108	0	*C. albicans*
**59**	M	66	BM	No	Ex	No	3040	0	*C. albicans*
**60**	F	66	BM	No	Yes	Mild	20	0	*C. albicans*
**61**	M	60	PAL	No	Yes	Mild	477	16	*C. albicans*
**62**	F	78	BM	Yes	Yes	No	150	41	*C. albicans*
**63**	F	60	TON	Yes	Yes	Moderate	40	0	*C. abicans*
**64**	M	38	TON	No	Ex	Severe	120	60	*C. albicans*
**65**	M	47	BM	No	Yes	Moderate	180	5	*C. albicans*
**66**	F	48	PAL	No	Yes	Moderate	43	2	*C. albicans*
**67**	M	81	BM	Yes	Ex	Severe	CSC	270120	*C. albicans* *C. tropicalis*
**68**	F	73	BM	No	Yes	Severe	0	0	None
**69**	F	56	TON	No	Yes	Severe	0	0	None
**70**	F	62	BM	No	No	Severe	0	0	None
**71**	F	56	FM	No	No	Severe	0	0	None
**72**	M	62	AR	No	No	Severe	0	0	None
**73**	F	61	TON	No	No	Moderate	0	0	None
**74**	F	61	PAL	No	No	Severe	0	0	None
**75**	F	40	AR	No	No	No	0	0	None
**76**	M	51	GIN	No	No	No	0	0	None
**77**	M	51	BM	No	No	Severe	0	0	None
**78**	M	54	BM	No	No	Severe	0	0	None

a Both of these patients with CL went on to develop squamous cell carcinoma at the lesional site within a two-year follow up period.

Abbreviations; CL, *Candida* leukoplakia; NCL, Non-*Candida* leukoplakia; SCC, squamous cell carcinoma; SC, semi-confluent growth (approx. 1000 cfu per swab or per ml oral wash); C, confluent (approx. 5000 cfu per swab or per ml oral wash); BM, buccal mucosa; TON, tongue, PAL, palate, AR, alveolar ridge; FM, floor of mouth; GIN, gingivae.

Oral rinse samples were also taken from a control group of 110 healthy volunteers recruited from the DDUH Accident and Emergency Department for comparison. Of these, 56 (50.9%) were male and 54 (49.1%) were female, the mean age was 54.1 years (range 29–78 years) and none had oral leukoplakia. These samples were taken during the same time period as the leukoplakia samples (i.e. December 2006-March 2009). Twenty-one healthy volunteers were smokers (19.0%) and six (5.5%) wore upper dentures.

Patients with leukoplakia and healthy volunteers were excluded from the study if they met any of the following criteria: pregnancy or lactation, diabetes or asthma, steroid treatment during the last year, antibiotic or anti-fungal treatment in the previous six months.

### Histopathological diagnosis of CL and NCL lesions

A DDUH oral surgeon performed incisional lesional biopsies. Biopsy samples were fixed in 10% (v/v) neutral buffered formalin (4% (v/v) formaldehyde). Histopathological investigation of biopsy samples was performed at the Central Pathology Laboratory at St. James's Hospital, Dublin. Fixed tissues were dehydrated with 70–100% (v/v) ethanol, cleared with xylene and embedded in paraffin wax. Tissue samples were then cut into 6–8 µm thick sections and routinely stained with haematoxylin and eosin. As candidal hyphae are poorly stained by hematoxylin/eosin, the periodic acid-Schiff (PAS) stain was also used, as hyphal structures stain strongly with PAS [18]. An oral and maxillofacial pathologist made diagnoses based on the histological features of each specimen.

### Sampling of the oral cavity and leukoplakia lesions

Oral rinse samples were performed as described previously [19]. *Candida* isolates were recovered by culture following incubation at 37°C for 48 h on the chromogenic culture medium CHROMagar Candida^TM^ (CHROMagar Company, Paris, France). This medium permits the presumptive identification of several clinically important *Candida* species including *C. albicans* based on colony colour and morphology [20].

Swab samples were taken using sterile cotton transport swabs and then transferred to alginate gel transport medium (Venturi Transystem, Copan Italia s.p.a, Brescia, Italy). Swab samples were taken by rubbing the entire surfaces of the lesion for 30 s; the swabbed areas sampled depended on the size of the lesion. Swab samples were plated within 2 h on to CHROMagar Candida^TM^ medium and incubated as above.

### Identification of *Candida* isolates

Following incubation, CHROMagar Candida^TM^ plates were examined, and the relative abundance of each colony type present was recorded. Selected examples of each colony type were chosen for detailed analysis. Isolates were initially presumptively identified on the basis of colony color and morphology [20], [21] and were definitively identified by substrate assimilation profiles using the API ID 32C yeast identification system (BioMérieux, Marcy l′Etoile, France) [22].

### DNA extraction

Genomic DNA from *C. albicans* isolates was extracted using the Qiagen DNeasy blood and tissue kit (Qiagen Crawley, West Sussex, UK) according to the manufacturer's instructions.

### ABC genotyping

Template DNAs extracted from isolates were assigned to genotypes A, B or C based on the differential PCR amplification of the 25S rRNA gene, as previously described [14].

### MLST

Twenty-five *C. albicans* isolates from CL lesions and 19 isolates from NCL lesions, from separate patients in each case (with the exception of two isolates, CL12 and CL122 which were recovered from separate CL lesions in patient 12), were subjected to MLST as described previously [12]. For comparison, MLST was also performed on 34 oral carriage (OC) *C. albicans* isolates recovered from oral rinse samples of 34 separate control healthy subjects attending the DDUH Accident and Emergency Department. All DNA sequencing reactions were performed commercially by Source Bioscience LifeSciences (Dublin, Ireland) using ABI 3730*xl* DNA analysers and dye-labelled terminators (Applied Biosystems, Foster City, CA, USA). Sequences were analysed using BioNumerics version 5.0 software (Applied Maths NV, Saint-Martens-Latem, Belgium) and the online *C. albicans* MLST database (http://calbicans.mlst.net). All MLST data generated in the present study have been deposited in the *C. albicans* MLST database (http://calbicans.mlst.net).

The genetic relatedness of the isolates investigated in the present study to each other and to selected strains from the online MLST database was evaluated using the software program MEGA version 5 to generate dendrograms based on the unweighted-pair group method with arithmetic averages (UPGMA) [23]. The UPGMA clustering algorithm defines MLST clades that correlate well with those previously defined by DNA fingerprinting using the *C. albicans*-specific repetitive sequence-containing Ca3 probe [24].

The online Based Upon Related (eBURST) algorithm (http://eburst.mlst.net) was used to subdivide MLST allelic profile datasets into hypothetical non-overlapping groups of clonal complexes (CCs) composed of a single putative founder diploid sequence type (DST) and its closely related descendant DSTs [25], [26].

### Statistical Analyses

Statistical analyses such as Fisher's exact tests and two-tailed Student's *t*-tests for independent sample proportions were carried out using Graphpad software (Graphpad Software Inc., CA, USA). A power analysis was undertaken based on the comparison of two independent samples. The parameters for this one sided test used a desired power value of 75% with a α value 0.05 (http://stat.ubc.ca/~rollin/stats/ssize/b2.html).

## Results

### Oral leukoplakia and healthy control subjects

Clinical details of the oral leukoplakia patients are shown in Table 1. Seventy-eight patients with oral leukoplakia were enrolled in the study, of whom 31/78 (39.7%) had histopathologically-defined CL. Biopsies from the remaining 47 patients (60.3%) were classified as NCL following histopathological investigation (Table 1). None of the healthy subjects included in the study had clinical signs of oral leukoplakia. The buccal mucosa was the most prevalent lesional site in the CL group (22/31; 71%), followed by the tongue (4/31; 12.9%), palate (3/31; 9.7%), gingivae (1/31; 3.2%) and alveolar ridge (1/31; 3.2%) (Table 1). The buccal mucosa was also the most prevalent lesional site in the NCL group (21/47; 44.7%), followed by the tongue (9/47; 19.1%), alveolar ridge (6/47; 12.8%), palate (5/47; 10.6%), the floor of the mouth (4/47; 8.5%) and gingivae (2/47; 4.3%) (Table 1).

### *Candida* detection by oral rinse sampling

*Candida* isolates were recovered from oral rinse samples in 55/78 oral leukoplakia patients (70.5%). *Candida albicans* was the most prevalent *Candida* species recovered and was isolated from all 55 *Candida*-positive oral leukoplakia patients, either alone (48 patients) or in combination with other *Candida* species or *Saccharomyces cerevisiae* (seven patients) (Table 1). Oral rinse sampling of 24/31 (77.4%) patients with CL yielded *Candida* species, all of whom harboured *C. albicans*, either alone (20 patients) or in combination with other *Candida* species or *S. cerevisiae* (four patients) (Table 1). Oral rinse sampling of 31/47 (66%) NCL patients yielded *Candida* species, all of whom harboured *C. albicans*, either alone (28 patients) or in combination with other *Candida* species (3 patients) (Table 1).

The average *Candida* cell density recovered by oral rinse sampling of the 55 *Candida*-positive patients was 719 cfu/ml (range 2 cfu/ml to approximately 6000 cfu/ml) (Table 1). Oral rinse sampling of 34/110 (30.9%) healthy control subjects yielded *C. albicans*. The median and mean average *Candida* cell density values recovered from these 34 *Candida*-positive controls was 18 and 32 cfu/ml, respectively (range 1–150 cfu/ml). No other yeast species were recovered from the healthy control subjects.

### *Candida* detection by lesional swab sampling

*Candida* species were recovered by swab sampling of oral leukoplakia lesions from 50/78 (64.1%) of the total oral leukoplakia patient cohort. *Candida albicans* was the predominant species recovered and was isolated from 48/50 (96%) *Candida*-positive patients either alone (42 patients) or in combination with other *Candida* species or *S. cerevisiae* (six patients). One patient (patient 49) yielded *C. krusei* only and another patient (patient 36) yielded *C. glabrata* only using the swab method. Both of these patients yielded *C. albicans* by oral rinse (Table 1).

Swab sampling of lesional tissues from 27/31 (87.1%) patients with CL yielded positive cultures for *Candida* species, all of which consisted of *C. albicans* either alone (23 patients) or in combination with other *Candida* species or *S. cerevisiae* (Table 1). Swab sampling of lesional tissues from 23/47 (48.9%) patients with NCL yielded positive cultures for *Candida* species, 21/23 (91.3%) of which consisted of *C. albicans* either alone (20 patients) or in combination with *C. tropicalis* (patient 67). One patient yielded *C. glabrata* only (patient 36) and one patient yielded *C. krusei* only (patient 49) (Table 1).

Interestingly, a comparison of oral rinse and lesional swab *Candida* culture data revealed that swab samples from oral lesions in eight patients [five CL patients (03, 07, 08, 09 and 28) and three NCL patients (34, 48 and 56)] were culture-positive for *Candida*, whereas the corresponding oral rinse samples were culture-negative (Table 1). The opposite was true for patient 40, from whom *Candida* was recovered by oral rinse, but not by lesional swabbing (Table 1).

### MLST analysis of *C*. *albicans*

Isolates from CL (n = 25), NCL (n = 19) and healthy controls (n = 34), were analysed with the consensus *C*. *albicans* MLST scheme [12]. MLST resulted in a dataset of 2,883 bp for each isolate. Single nucleotide polymorphisms (SNPs) were identified in 84 of the 2,883 bp (2.9%) analysed. The *AAT1a* gene yielded 11 SNPs, (13.1%) the *ACC1* gene yielded six (7.1%) SNPs, the *ADP1* gene yielded 14 (16.7%) SNPs, the *MPIb* gene yielded 12 (14.2%) SNPs, the *SYA1* gene also yielded 11 (13.1%) SNPs, the *VPS13* gene yielded 15 (17.9%) SNPs and the *ZWF1* gene yielded 15 (17.9%) SNPs.

**Figure 1:**
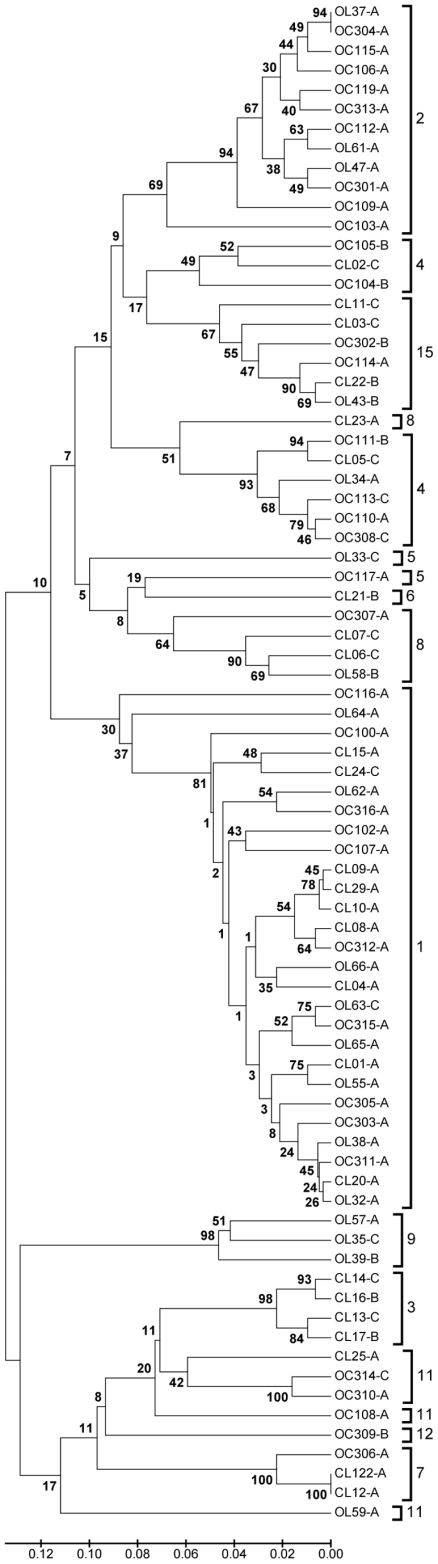
UPGMA dendrogram depicting the genetic relatedness of all *C.* *albicans* isolates subjected to MLST and ABC genotyping analysis in the present study. Individual isolates recovered from CL (n = 25) and NCL (n = 19) patients are indicated with the letters CL and OL in the isolate names, respectively. The numeral part of the isolate name refers to the corresponding patient numbers as shown in Table 1, column 1. Isolates recovered from age and sex-matched healthy control patients (n = 34) by oral rinse are indicated using the letters OC in the isolate name. Hyphenated letters A, B or C following each isolate name indicate ABC genotypes. The scale bar indicates p- distance. Distinct MLST clades are indicated by separated square parenthesis to the right of the isolate names and labelled with previously designated clade numbers [17]. Numbers at clade branches indicate bootstrap support levels, based on 1000 replications. Overall, the population analysis based on MLST suggests no clonal enrichment of *C. albicans* isolates recovered from CL or NCL lesions. Isolates recovered from CL patients are distributed among eight clades, isolates from NCL patients are distributed among eight clades and isolates from healthy carriers are distributed among nine clades, with clade 1 predominating in all three groups.

New allelic profiles and DSTs were identified in 56 isolates (Table 2). Of the seven loci investigated for each isolate, five new alleles were identified including four new *ZWF1b* alleles, and one *MPIb* allele. Only two DSTs were identified in duplicate; the newly identified DST2108 was identified in an isolate (OL37) recovered from an NCL lesion and in an isolate (OC304) recovered from a healthy individual. Another newly identified DST, 2105, was identified in isolates CL12 and CL122, however, these isolates were recovered from separate CL lesions in the same patient, patient 12 (Table 2 and Figure 1).

**Table 2:** MLST DSTs, allelic profiles and ABC genotypes of *C. albicans* isolates recovered from OL patients and healthy oral carriers.

Isolate a	Source	DST	MLST loci							MLST clade^b^	CC	Htz	ABC Genotype c
			*AAT*	*ACC*	*ADP1*	*MPI*	*SYA*	*VPS13*	*ZWF*				
**CL10**	CL	1235	2	2	2	31	2	24	5	1	1	14	A
**CL09**	CL	1234	2	2	2	27	2	24	5	1	1	14	A
**CL29**	CL	1659	2	2	2	2	2	24	5	1	1	15	A
**CL01**	CL	408	2	5	5	2	2	20	5	1	1	17	A
**CL20**	CL	1400	2	5	5	31	2	6	5	1	1	22	A
**CL08** d	CL	1239	2	23	5	2	2	63	5	1	S	18	A
**CL15**	CL	1237	73	5	5	9	2	127	20	1	S	7	A
**CL23**	CL	228	8	5	20	6	43	69	22	8	23	14	A
**CL25**	CL	538	62	12	21	1	6	30	4	11	5	23	A
**CL12**	CL	2105	6	3	37	2	38	46	237	7	16	27	A
**CL122**	CL	2105	6	3	37	2	38	46	237	7	16	27	A
**CL04**	CL	149	2	5	5	2	2	21	20	1	1	18	A
**CL17**	CL	1240	13	10	15	24	50	37	15	3	4	21	B
**CL21**	CL	1294	21	26	14	18	72	86	84	6	8	21	B
**CL22**	CL	1682	4	31	6	4	61	15	201	15	58	11	B
**CL16**	CL	2106	13	10	15	4	7	32	15	3	4	21	B
**CL24**	CL	79	2	5	5	9	2	6	5	1	1	17	C
**CL13**	CL	1236	13	32	15	6	7	140	15	3	4	28	C
**CL14** d	CL	1681	13	10	83	6	84	32	15	3	S	19	C
**CL05**	CL	1232	14	3	6	4	56	3	8	4	S	7	C
**CL02**	CL	1432	4	26	61	4	34	60	202	4	S	8	C
**CL06**	CL	1233	33	7	38	31	78	122	15	8	S	19	C
**CL07**	CL	1680	28	14	38	102	31	47	15	8	S	17	C
**CL03**	CL	1431	4	35	6	4	58	15	200	15	S	6	C
**CL11**	CL	2107	4	13	6	4	61	29	201	15	30	12	C
**OL62**	NCL	1648	8	3	2	4	2	6	5	1	1	9	A
**OL65**	NCL	1047	8	2	5	2	2	6	5	1	1	16	A
**OL66**	NCL	1651	2	2	5	2	2	63	20	1	1	8	A
**OL32**	NCL	69	2	5	5	2	2	6	5	1	1	23	A
**OL64**	NCL	1650	3	3	5	3	57	3	6	1	10	10	A
**OL47**	NCL	1238	35	2	4	4	49	4	4	2	2	3	A
**OL61**	NCL	1496	35	2	4	4	49	26	4	2	2	7	A
**OL34**	NCL	481	8	14	8	4	2	3	8	4	3	4	A
**OL57**	NCL	1683	62	3	3	3	26	16	95	9	6	19	A
**OL59**	NCL	1685	67	3	10	1	6	8	20	11	S	8	A
**OL37**	NCL	2108	35	4	4	4	4	41	4	2	3	19	A
**OL38**	NCL	37	2	5	5	2	2	21	5	1	1	22	A
**OL55**	NCL	1224	2	2	2	2	2	20	5	1	1	16	A
**OL58**	NCL	1684	28	14	38	2	106	122	15	8	S	22	B
**OL39**	NCL	1429	3	3	3	3	67	16	95	9	S	13	B
**OL43**	NCL	2109	4	82	6	4	61	32	201	15	S	11	B
**OL63**	NCL	1649	8	3	2	2	2	6	5	1	1	16	C
**OL33**	NCL	1686	11	8	4	3	7	19	4	5	S	23	C
**OL35**	NCL	1652	32	3	43	3	3	32	94	9	S	15	C
**OC102**	OC	1660	3	5	5	2	2	76	5	1	1	18	A
**OC100**	OC	1428	2	23	5	102	2	20	20	1	S	10	A
**OC107**	OC	1664	4	5	6	2	2	106	5	1	S	16	A
**OC116**	OC	1673	2	3	10	2	2	94	2	1	S	16	A
**OC106**	OC	1663	4	5	4	4	139	26	4	2	2	11	A
**OC119**	OC	275	35	2	4	4	4	4	4	2	2	8	A
**OC103**	OC	1661	40	24	41	21	4	76	27	2	S	13	A
**OC109**	OC	1666	4	2	14	4	139	41	67	2	S	7	A
**OC112**	OC	1669	36	2	6	4	49	41	4	2	S	4	A
**OC110**	OC	1667	8	14	8	4	56	10	8	4	3	9	A
**OC115**	OC	1672	4	60	6	4	4	41	4	2	S	11	A
**OC117**	OC	1674	13	3	6	34	62	8	47	5	S	16	A
**OC108**	OC	1665	5	27	37	4	34	105	12	11	S	20	A
**OC114**	OC	1671	4	19	6	4	61	15	201	15	58	8	A
**OC301**	OC	2110	35	4	4	4	49	4	4	2	3	11	A
**OC303**	OC	2111	2	3	6	2	2	5	5	1	1	19	A
**OC304**	OC	2108	35	4	4	4	4	41	4	2	3	19	A
**OC305**	OC	2112	2	5	5	2	2	5	12	1	1	17	A
**OC306**	OC	492	6	3	37	2	38	32	12	7	16	20	A
**OC307**	OC	1118	55	3	4	54	6	45	15	8	12	11	A
**OC310**	OC	2113	60	7	21	1	50	11	15	11	11	15	A
**OC311**	OC	1133	2	5	5	2	2	5	5	1	1	22	A
**OC312**	OC	24	2	5	5	2	2	24	5	1	1	18	A
**OC313**	OC	2114	35	4	4	4	4	4	26	2	3	15	A
**OC315**	OC	171	8	3	6	2	2	6	5	1	1	16	A
**OC316**	OC	444	2	5	5	4	2	6	5	1	1	17	A
**OC105**	OC	659	11	26	6	4	34	60	119	4	9	10	B
**OC104**	OC	1662	3	26	6	4	34	60	55	4	S	4	B
**OC111**	OC	1668	14	14	30	4	56	3	8	4	S	8	B
**OC302**	OC	2115	4	35	6	4	61	15	5	15	62	12	B
**OC309**	OC	299	4	17	21	19	27	83	22	12	7	15	B
**OC113**	OC	1670	8	7	6	4	56	3	118	4	S	5	C
**OC308**	OC	924	8	14	6	4	7	3	8	4	2	9	C
**OC314**	OC	2116	60	7	21	1	7	55	15	11	11	26	C

a With regard to isolates recovered from CL and NCL patients, the numeral part of the isolate name refers to the corresponding patient numbers shown in Table 1, column one (e.g. isolate CL10 was recovered from patient 10). Isolates CL12 and CL122 were recovered from two separate CL lesions in patient 12. CL and NCL isolates were recovered from lesional swabs, whereas OC isolates were recovered from oral rinse samples.

b MLST clades defined according to Odds *et al*. [17].

c ABC genotypes were assigned based on the presence or absence of an intron in the 25S rRNA gene [14].

d Two CL patients included in this study (patients 08 and 14, Table 1) developed squamous cell carcinoma at the lesional site within a two-year follow up period. Isolate CL14 (patient 14) and isolate CL08 (patient 8) belonged to ABC genotypes C and A, respectively.

Abbreviations: DST, diploid sequence type; CC, clonal complex; Htz, number of heterozygous sites; CL, *Candida* leukoplakia; NCL, non-*Candida* leukoplakia; OC, oral carriage; S, singleton.

A UPGMA dendrogram was constructed using the MLST allelic profile data resulting from 44 isolates recovered from oral leukoplakia lesions, 34 OC isolates and 42 isolates representative of previously documented MLST clades previously included in the *C. albicans* MLST database (data not shown), to determine the genetic relatedness of isolates in the present study to those previously included in the MLST database, and to assign newly identified DSTs to clades. This tree identified the presence of distinct MLST clades as previously described [17]. A smaller UPGMA dendrogram was constructed based on the concatenated MLST SNPs of only the 78 isolates included in the current study (Figure 1). This dendrogram shows that isolates recovered from both CL and NCL patient groups are distributed amongst 11 distinct MLST clades, and OC isolates are distributed amongst nine distinct MLST clades, with clade 1 predominant in all three groups of patients (Figure 1 and Table 2). Of the 78 isolates investigated by MLST, 27/78 (34.6%) belonged to *C. albicans* MLST clade 1, consisting of nine CL isolates, eight NCL isolates and 10 OC isolates. A further 12/78 (15.4%) isolates belonged to MLST clade 2, of which nine were OC isolates and three were NCL isolates. There was an absence of CL isolates in clade 2. The third most predominant clade was clade 4, which contained 9/78 (11.5%) isolates including six OC isolates, two CL isolates and one NCL isolate. Interestingly, all four isolates belonging to clade 3 in the present study were recovered from CL lesions, and all three isolates belonging to clade 9 in the present study were recovered from NCL lesions (Figure 1 and Table 2).

The level of heterozygosity amongst the 84 polymorphic nucleotides were determined for the *C. albicans* isolates investigated in the present study, as significant losses of heterozygosity can be associated with minor genetic switches or microvariation [15], [27]. The average number of heterozygous sites in isolates recovered from CL lesions was 16.9±6.2 sites compared to 13.9±14.1 sites in isolates recovered from NCL lesions and 13.4±5.4 sites in the OC isolates recovered from healthy patients (Table 2). These differences are most likely the result of the minor differences in clade prevalence amongst isolates from each patient group.

According to eBURST analysis, an alternative method of predicting evolutionary patterns and founding genotypes, the most predominant clonal complex (CC) identified amongst 21/78 (26.9%) isolates from the present study was CC1. CC1 isolates were recovered from seven CL patients, seven NCL patients and seven OC subjects (Table 2). Five isolates belonged to CC2 and six to CC3 (Table 2). Singletons that were not assigned to any CCs were identified in isolates recovered from 8/25 (32%) CL patients, 6/19 (31.6%) NCL patients and 12/34 (35.3%) OC isolates (Table 2).

### ABC genotyping of *C. albicans* isolates

The same 78 isolates that were investigated by MLST were subjected to ABC genotyping, including isolates from CL (n = 25), NCL (n = 19), and OC (n = 34). Genotype A was the most common genotype and was identified in 51/78 (65.4%) isolates. Twelve isolates (15.4%) were identified as genotype B and 15 (19.2%) were identified as genotype C (Table 2). Of the 34 OC isolates, 26 (76.5%) were identified as genotype A, five were identified as genotype B (14.7%), and the remaining three were genotype C (8.8%). In contrast, only 25/44 (56.8%) oral leukoplakia isolates (12 CL and 13 NCL) belonged to genotype A (Table 3). Genotype B was identified in seven (15.9%) oral leukoplakia isolates (four CL and three NCL). Interestingly, 12/44 (27.3%) oral leukoplakia isolates were identified as genotype C, (nine CL and three NCL). The proportions of genotypes A–C among CL and OC isolates were significantly different (P<0.02 with Fisher's exact test). Isolates from CL lesions were significantly enriched with genotype C 9/25 (36%) when compared to OC isolates (3/34, 8.8%) from healthy carriers (P<0.05 with two sample Student's *t*-test). Due to the small number of CL isolates available for investigation in the present study, a power analysis was undertaken to determine if the sample size was sufficient to reliably detect this increased prevalence of genotype C. Using a desired power value of 75% with an α value 0.05, a sample size of 25 was indicated for each sample group. As 25 CL isolates and 34 OC isolates were included in the study, the results of the power analysis indicated that sufficient isolates were investigated to support the significance of the observed increased prevalence of genotype C isolates amongst CL isolates relative to OC isolates.

**Table 3:** Distribution of ABC genotypes in isolates recovered from oral leukoplakia patients and healthy carriers.

Patient Group	Number of isolates	Genotypes identified a n *(%)*
CL	25	A = 12 (48)B = 4 (16)C = 9 (36)
NCL	19	A = 13 (68.4)B = 3 (15.8)C = 3 (15.8)
OC	34	A = 26 (76.5)B = 5 (14.7)C = 3 (8.8)

a The prevalence of genotype A and genotype C isolates differed significantly between the CL and OC groups (P<0.02).

Abbreviations: CL, *Candida* leukoplakia; NCL, non-*Candida* leukoplakia; OC, oral carriage.

## Discussion

The present study observed that *Candida* isolates were more frequently recovered by oral rinse sampling of patients with oral leukoplakia (70.5% positive) than of healthy controls (30.9% positive). Unsurprisingly, *C. albicans* was the most frequently identified *Candida* species, and was recovered from all *Candida*-positive oral leukoplakia patients either alone, or in combination with other species. No *Candida* species other than *C. albicans* were recovered from the healthy control population. Oral leukoplakia patients were histopathologically-diagnosed as having CL or NCL in the present study. *Candida* species were recovered from swab sampling of 64.1% of the oral leukoplakia patients, and in 87.1% of patients with CL. Interestingly, comparative oral rinse and lesional swab sampling methods revealed that in eight oral leukoplakia patients, lesional swab yields of *Candida* species did not correlate with corresponding oral rinse sampling *Candida* yields (Table 1). It is possible that the oral rinse sampling method, and to a lesser extent swab sampling, was not vigorous enough to dislodge strongly adherent and/or invasive *Candida* blastospores or hyphae from these CL lesions.

MLST is a very effective tool for *C. albicans* population analyses [11]. Overall, the population analysis based on MLST suggests that no clonal enrichment exists in isolates recovered from CL or NCL lesions. The isolates recovered from both CL and NCL patient groups are distributed amongst 11 distinct MLST clades, and OC isolates are distributed amongst nine distinct MLST clades. Clade 1 predominated in all three groups of patients with 27 isolates (34.6%) belonged to this clade. Twelve isolates (15.4%) belonged to MLST clade 2, and nine (11.5%) isolates belonged to MLST clade 4. This study therefore supports previous findings [17], [28], that clades 1, 2 and 4 comprise more than the 2/3 of isolates in the MLST database.

Interestingly, there was an absence of CL isolates in clade 2, whereas 9/12 (75%) clade 2 isolates consisted of OC isolates representing 26.5% (9/34) of all OC isolates included in the study. All four isolates belonging to clade 3 in the present study were recovered from CL lesions. Furthermore, all three isolates belonging to clade 9 in the present study were recovered from NCL lesions. It is likely that increasing the numbers of CL, NCL and OC isolates in larger future studies would result in the diversification of these clades (Fiure 1).

Currently, there are 2,218 *C. albicans* isolate profiles in the MLST database (http://calbicans.mlst.net/) comprising 2,086 unique allelic profiles and their corresponding DSTs (date accessed: 10^th^ April 2013), of which 373 (17.9%) isolates are described as oral isolates comprising 290 distinct DSTs. According to the eBURST algorithm, these oral isolate DSTs can be divided into 24 different CCs, with CC1 predominating. The remaining 131/290 (45.2%) DSTs are classified as singletons. In the present study, analysis of 78 *C. albicans* MLST profiles using the eBURST algorithm showed that 50 isolates were distributed among 17 CCs, with CC1 predominating. Twenty-six (33.3%) isolates were assigned as singletons. This suggests that, like the UPGMA clade-based method, eBURST identifies no significant clonal enrichment in isolates recovered from oral leukoplakia patients. Overall, our MLST data correlates with previous biotyping studies [10] which failed to find clonal distinction among *C. albicans* strains recovered from CL lesions.

ABC genotyping of *C. albicans* isolates was included in the present study to provide further isolate discriminatory data. Similarly to previous studies [13], [16], [17], [29] genotype A predominated in all groups examined, the majority (92.6%) of isolates belonging to MLST clade 1 were identified as genotype A. Surprisingly, genotype C was identified more frequently in the current study than in the aforementioned studies, particularly in isolates from oral leukoplakia lesions (Table 3), being identified in 9/25 (36%) and 3/19 (15.8%) of isolates recovered from CL and NCL lesions, respectively (Table 3). In contrast, only three genotype C isolates were identified among the 34 (8.8%) OC isolates investigated. However, the MLST data clearly did not support any suggestion of clonal enrichment (Figure 1 and Table 2). It is important to emphasise that MLST has been shown to be an exquisitely informative tool for inferring clonal relationships between *C. albicans* isolates [17] in contrast to ABC genotyping, which examines only one locus and can only separate isolates into three groups. ABC genotypes A and B are determined by the absence or presence of an intron in the 25S rRNA gene, respectively. Genotype C is a hybrid of the other two genotypes, containing the intron in only one allele. On balance, it is unlikely that the observed genotype C enrichment among isolates from leukoplakia lesions reflects a specific clonal enrichment. This suggestion is supported by the findings that ABC genotype C was identified in *Candida* leukoplakia isolates belonging to MLST clades 1, 3, 4, 8 & 15, and in non-*Candida* leukoplakia isolates belonging to MLST clades 1, 5 & 9.

The oral leukoplakia isolates investigated in the present study might represent a geographical clonal population of *C. albicans* from Ireland. However, this seems unlikely as the OC control isolates were also from the same geographical location but exhibited a different ABC genotype distribution (Table 3). Further investigations based on a larger patient numbers are required and should include individuals from disparate geographical locations and ethnic backgrounds in order to decipher this phenomenon further. Additional investigations might involve comparative SNP microarray or whole genome sequence analysis of several CL and NCL isolates from each ABC genotype.

Very few previous studies actually correlated *C. albicans* ABC genotypes with specific medical conditions. Most previous studies that used ABC genotyping did so as a secondary typing method in combination with another, more discriminatory typing method such as microsatellite typing or MLST. *Candida albicans* ABC genotypes tend to be more often associated with particular MLST clades rather than with particular medical conditions [17]. For example, one previous study identified an enrichment of MLST clade 1 *C. albicans* isolates recovered from sub-gingival sites in patients with untreated periodontal disease; 21/31 isolates investigated belonged to MLST clade 1 and all were found to belong to ABC genotype A [19]. This finding was not unusual, as many previous studies have shown that MLST clade 1 consists predominantly of ABC genotype A isolates [17]. No ABC genotype C isolates were identified in the periodontal study. In a separate study on 14 oral *C. albicans*-positive patients with Autoimmune Polyendocrinopathy-Candidiasis-Ectodermal Dystrophy (APECED) syndrome, a rare genetic disease characterised by autoimmunity to endocrine organs, ectodermal disorders and chronic mucocutaneous candidiasis, 9/14 patients yielded genotype A isolates, six of which belonged to MLST clade 1 [30]. One patient yielded genotype B isolates (MLST clade 8) and four patients yielded genotype C isolates, two of which belonged to MLST clade 4, one to clade 15, whereas the remaining isolate was a singleton.

Based mainly on case reports, the potential for malignant transformation of CL is well recognised [31], [32], and *Candida* has previously been implicated as having an aetiological role in initiation of carcinoma in leukoplakia lesions associated with acetylaldehyde production [33], [34]. A recent study reported that *C. albicans* adhesion, tissue invasion and damage of epithelial cells is influenced by a combination of fungal morphology and activity and epithelial cell type and stage of differentiation, suggesting that epithelial cells differ in their susceptibility to *C. albians* [35]. This study also showed that *C. albicans* can invade oral epithelial cells by induced endocytosis and by active penetration.

In the present study two cases of malignant transformation occurred in diagnosed CL lesions, one infected with an MLST clade 1, ABC genotype A isolate and the other infected with an MLST clade 3, ABC genotype C isolate, during the follow up period of two years (Table 1). Overall, the results of the present study do not show enrichment of specific *C. albicans* clonal lineages among isolates recovered from CL lesions although the apparent increased association of ABC genotype C isolates with CL lesions is intriguing. Our findings suggest that *C. albicans* invades leukoplakia lesions giving rise to *Candida* leukoplakia and that lesional factors are probably more significant than the *C. albicans* lineage. Larger studies are required to further investigate the role of *Candida* in CL.
